# Potential Usage of Hybrid Polymers Binders Based on Fly Ash with the Addition of PVA with Satisfying Mechanical and Radiological Properties

**DOI:** 10.3390/gels7040270

**Published:** 2021-12-16

**Authors:** Miljana Mirković, Ljiljana Kljajević, Sabina Dolenec, Miloš Nenadović, Vladimir Pavlović, Milica Rajačić, Snežana Nenadović

**Affiliations:** 1 Department of Materials, “VINČA” Institute of Nuclear Sciences—National Institute of the Republic of Serbia, University of Belgrade, Mike Petrovića Alasa 12–14, 11000 Belgrade, Serbia; ljiljana@vinca.rs (L.K.); msneza@vinca.rs (S.N.); 2 Slovenian National Building and Civil Engineering Institute, Dimičeva Ulica 12, 1000 Ljubljana, Slovenia; sabina.dolenec@zag.si; 3 Department of Atomics Physics, “VINČA” Institute of Nuclear Sciences—National Institute of the Republic of Serbia, University of Belgrade, Mike Petrovića Alasa 12–14, 11000 Belgrade, Serbia; milosn@vinca.rs; 4 Faculty of Agriculture, University of Belgrade, Nemanjina 6, 11000 Belgrade, Serbia; vladimirboskopavlovic@gmail.com; 5 Department of Radiation and Environmental Protection, “VINČA” Institute of Nuclear Sciences—National Institute of the Republic of Serbia, University of Belgrade, Mike Petrovića Alasa 12–14, 11000 Belgrade, Serbia; milica100@vinca.rs

**Keywords:** geopolymer gel, inorganic binders, hybrid materials, fly ash, radiological properties, strength

## Abstract

Since recycled technologies usage is mandatory for environmental safety, and in this regard, it is important to examine new materials that can be used in construction and are primarily produced from fly ash. In addition to characteristics such as hardness and compressive strength, the given materials must also be radiologically and environmentally safe. The main concept of engineered geopolymer gel composites based on fly ash residues is focused on developing binder materials via gel formation processes that can replace ordinary cement materials. This study is unique in researching the potential use of fly ash from the Nikola Tesla thermal power plant in Serbia, where the hybrid geopolymeric materials synthesized from fly ash are experimentally examined with the addition 1 wt% and 2 wt% of polyvinyl alcohol (PVA). This paper aims to investigate the structural, morphological, mechanical, and radiological properties of hybrid materials with the addition of PVA and without additive in the period of ageing for 28 days at room temperature. The phase composition was investigated using X-ray powder diffraction (XRPD) analysis, while morphological characteristics of these materials were examined using scanning electron microscopy and energy dispersive X-ray analysis (SEM-EDS). Vibrational spectra of obtained samples are investigated using diffuse reflectance infrared Fourier transform (DRIFT) and Fourier transform infrared (FTIR) techniques. The hardness and compressive strength are also examined, indicating that the 1 wt% addition in geopolymeric matrix results in the best mechanical properties. Radiological measurements of investigated all geopolymer samples show decreasing activity concentrations of radionuclides for 50% compared to fly ash.

## 1. Introduction

In the past two decades, alkaline-activated materials as binders have attracted much attention to researchers since raw materials are sources for their development. The importance of the recycling-reuse of raw materials concept has been mandatory nowadays due to environmental protection since worldwide trends indicate a large cement and concrete consumption in the building sector. Geopolymers represent alkali-activated materials compared to Portland cement, have satisfactory mechanical properties. However, there is a constant search for better performance, especially in terms of hardness and compressive strength and the elasticity of these materials [1,2,3]. It is unequivocally clear that the synthesis of geopolymer materials is economically and ecologically more profitable, given that secondary raw materials are used as the base precursor. Their synthesis also leads to a significantly lower release of carbon dioxide and energy consumption [4,5,6]. Due to such needs, organic components are most often added to the gel geopolymer matrix, most often polyvinyl alcohol-PVA [3,7]. Polyvinyl alcohol (PVA) is a typical water-soluble synthetic polymer containing vinyl group [8,9]. Moreover, PVA is harmless and relatively environmentally friendly [10]. It has a carbon chain backbone with hydroxyl groups attached to the carbon atoms. These OH groups can be a source of hydrogen bonds and, hence, can assist in forming a polymer network with a geopolymer matrix [11].

The raw material is primarily fly ash with a high amount of silica and alumina activated with alkaline solution forming starting gel matrix with polymer bonds between silicon, aluminum, and oxygen [12,13]. From the structural point of view, it is semi-crystalline aluminosilicates with an amorphous microstructure [14]. Some novel studies of geopolymer materials show that elastic modulus and flexural strength are higher than cement composites using the same pressure. However, on the other hand, it has higher brittleness than cement [15,16,17,18]. One of the most critical limitations of geopolymers is brittle behavior. To improve the mechanical characteristics of geopolymer materials, it is necessary to introduce some additives to the matrix. The best results are achieved through the mixed organic and inorganic polymers [19]. Incorporation of nanoparticles, glass, and especially polypropylene, and polyvinyl alcohol additives in primary geopolymer gel results in the formation of hybrid materials with enhanced mechanical properties [14,20]. PVA as polymers has good elastic deformation properties, flexibility, acid and alkali corrosion resistance. Their addition in the geopolymer matrix can effectively improve the mechanical properties, deformation properties, and durability of geopolymer-based materials [21,22,23]. After dissolving in water, the polyvinyl alcohol produces a film with good deformation properties, toughness, and wear resistance [24]. Considering the excellent performance, PVA was used to modify the mechanical properties of geopolymer-based materials. The addition of PVA increased the compressive strength of the cement mortar by about 12% due to the chemical interaction between PVA and cement in the hydrated form [24,25].

Besides good mechanical properties, one of the important characteristics of polymers made of fly ash is radiological properties. The radionuclides, mainly ^226^Ra, ^232^Th, and ^40^K, present in the building materials such as cement, concrete, and geopolymer, upon decay, produce radiation fields to which all human beings are exposed [26,27,28]. The external exposure is caused by direct gamma radiation. In contrast, internal exposure, which is most important for humans living spaces, is caused by the inhalation of radon (Rn-222) and (Rn-220), which are Ra-226 and Th-232 decay products. Radon is the second leading cause of lung cancer after smoking, and because it is an inert gas, it can move relatively freely through porous media such as building materials. In most cases, the main part of indoor radon on the upper floors originates from building materials [29,30]. In one of the standard screening methods the dose caused by building materials is the use of an Activity Concentration Index (ACI or I_γ_), the value of which is calculated based on the concentrations of Ra-226, Th-232, and K-40 and it is related to the gamma radiation dose in a building over the typical outdoor exposure [31].

The main aim of the work is to synthesize geopolymer materials from fly ash as a starting material with 1wt% and 2 wt% PVA to investigate obtained materials’ morphological, structural, and radiological performances. The study’s novelty is amplified with changes in the radioactivity index from raw fly ash and in hybrid geopolymer, where changes are monitored and studied depending on the addition of PVA. Besides radiological properties, this study reveals the straightforward and ecologically safe synthesis method to obtain new hybrid geopolymer materials for potential further use. As Mirković et al. previously reported [32], fly ash from the Nikola Tesla power plant (FA01) showed appropriate characteristics for cement clinker preparation in both chemical and radiological terms. This study represents a continuation of the previously [32] mentioned one. We present the morphological, mechanical, and radiological characteristics of cement materials synthesized from a siliceous type of fly ash (FA01).

## 2. Results and Discussion

### 2.1. XRPD Results

**Figure 1:**
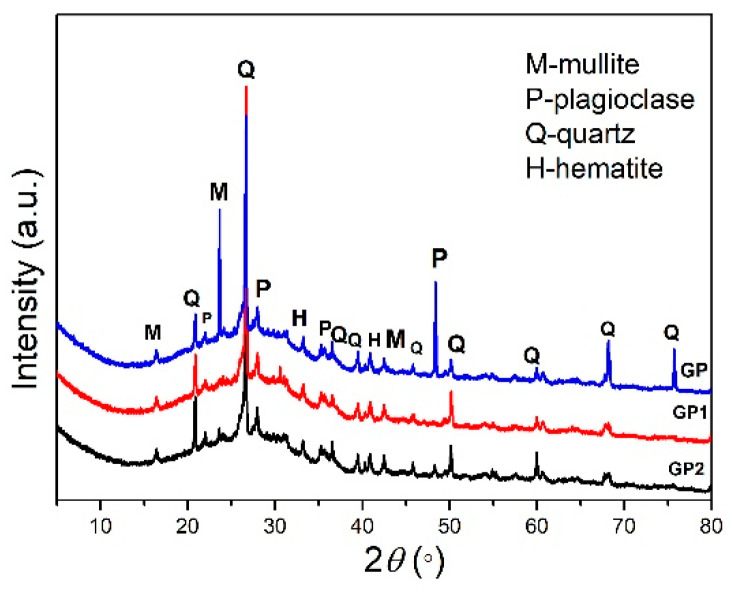
XRPD results of synthesized samples GP, GP1, and GP2.

Results of the mineralogical composition of synthesized samples are shown in Figure 1. The identified mineral phases in geopolymer samples do not differ much from the phases identified in fly ash—FA01 which contains: hematite, quartz, plagioclase, mullite as main phases which are presented in our previous research [32]. As the results show, the synthesized geopolymer (GP, GP1, and GP2) materials reveal crystal diffraction peaks of mullite, quartz, and plagioclase (albite) as main mineral forms in the geopolymer matrix. Intensities of mentioned phases in the GP sample show much higher intensities than in GP1 and GP2 samples. This increase in peak intensity was most likely due to the contribution due to the layered orientation of the mullite grain stacking (related to preferred orientation) which is a common occurrence during the sample preparation. Moreover, this also indicates that mentioned crystalline phases did not participate in geopolymerization reaction in GP sample [33]. Diffraction peaks intensities of similar crystalline phases in GP1 and GP2 samples are much lower than in GP sample. This can be explained by the process of hydration reaction and other chemical reactions that occur by geopolymerization creating an amorphous glassy mass wrapped in the matrix of quartz and mullite [34]. All diffraction patterns show broad background from about 20° to 40° 2*θ* respectively, indicating the formation of geopolymer matrix. The samples GP1 and GP2 have swelling diffraction peaks, which are characteristic of geopolymer reaction products [34], and it can be assumed that a higher amount of PVA addition in the geopolymer matrix leads to the better formation of the glassy matrix. The XRPD results indicate that the obtained GP, GP1, and GP2 materials show a semi-crystalline arrangement in amorphous geopolymer matrix [14].

### 2.2. DRIFT Analysis

**Figure 2:**
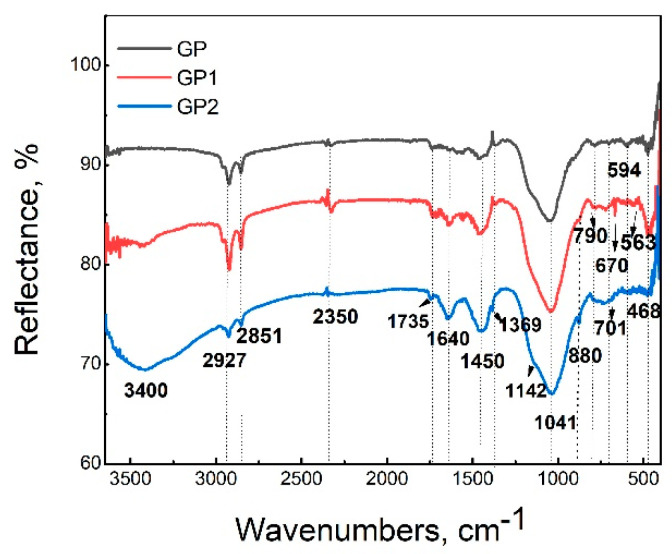
DRIFT spectra of GP, GP1, and GP2 samples.

Figure 2 shows the DRIFT spectra of geopolymer samples GP, GP1, and GP2. In geopolymer samples, large broadband at 3400 cm^−1^ is attributed to the stretching vibrations of O-H bonds and H-O-H bending vibrations of the interlayer adsorbed H_2_O molecules. This band is expressed in GP1 and particularly pronounced in GP2 due to added PVA in FA as a precursor [35,36]. The band at 1640 cm^−1^ is assigned to the surface of adsorbed water molecules [37,38]. The vibrational bands observed at 2851 and 2927 cm^−1^ refer to the stretching of C–H from alkyl groups, which are originated from PVA, while the peaks at 1735 cm^−1^ are due to the stretching of C=O and C–O from the acetate group remaining from PVA [39,40]. A peak observed in the spectra around 1450 cm^−1^ was assigned to the asymmetric CO_3_ stretching mode, which suggests the presence of sodium carbonate as a result of the reaction between excess sodium and atmospheric carbon dioxide [41]. A weak peak that was verified at 1142 cm^−1^ has been used as an assessment tool of poly (vinyl alcohol) structure because it is a semi-crystalline synthetic polymer able to form some domains depending on several process parameters [36]. However, this band is also presented in the FTIR spectrum of GP that does not contain PVA. When examining aluminosilicate raw materials for geopolymerization, this band is usually assigned to the asymmetric stretching vibrations of Si-O-Si bonds that are enriched by Al during geopolymerization and are displaced to lower wavenumbers [42,43]. This band overlapped with a broad characteristic band centered at 1045 cm^−1^. This peak is attributed to the Si-O stretching in tetrahedrons characterized by silicon bound to three bridging oxygen units and one non-bridging oxygen [44,45]; Si–O–Si symmetric or Al–O–Si asymmetric stretching mode. These structures are the building blocks of geopolymers [46,47]. These bands in the spectra of fly ash are centered at a higher wavelength [48]. As a result of the alkaline activation process of the FA01 as precursor material, the geopolymer samples were obtained. This strong band shifts toward a low wavenumber in the geopolymer samples. This demonstrates that a noticeable change in the microstructure occurs during the geopolymerization reaction, resulting in a new product with a different microstructure [38,49]. The Si–O bending bands are found at 880 cm^−1^. The peaks at 790 cm^−1^ and 468 cm^−1^ corresponded to quartz’s symmetric Si–O stretching vibrations [45]. According to the literature [50,51], vibration bands attributed to secondary building units made of joined SiO_4_ and AlO_4_ tetrahedral, forming variously membered rings in the 800–550 cm^−1^ region. Moreover, these bands in the region 800–560 cm^_1^ (790, 670, and 563 cm^−1^) are associated with Si-O-Al vibrations [52,53], and are more noticeable in geopolymers with a lower mass fraction of PVA (GP1).

### 2.3. SEM-EDS Analysis

**Figure 3:**
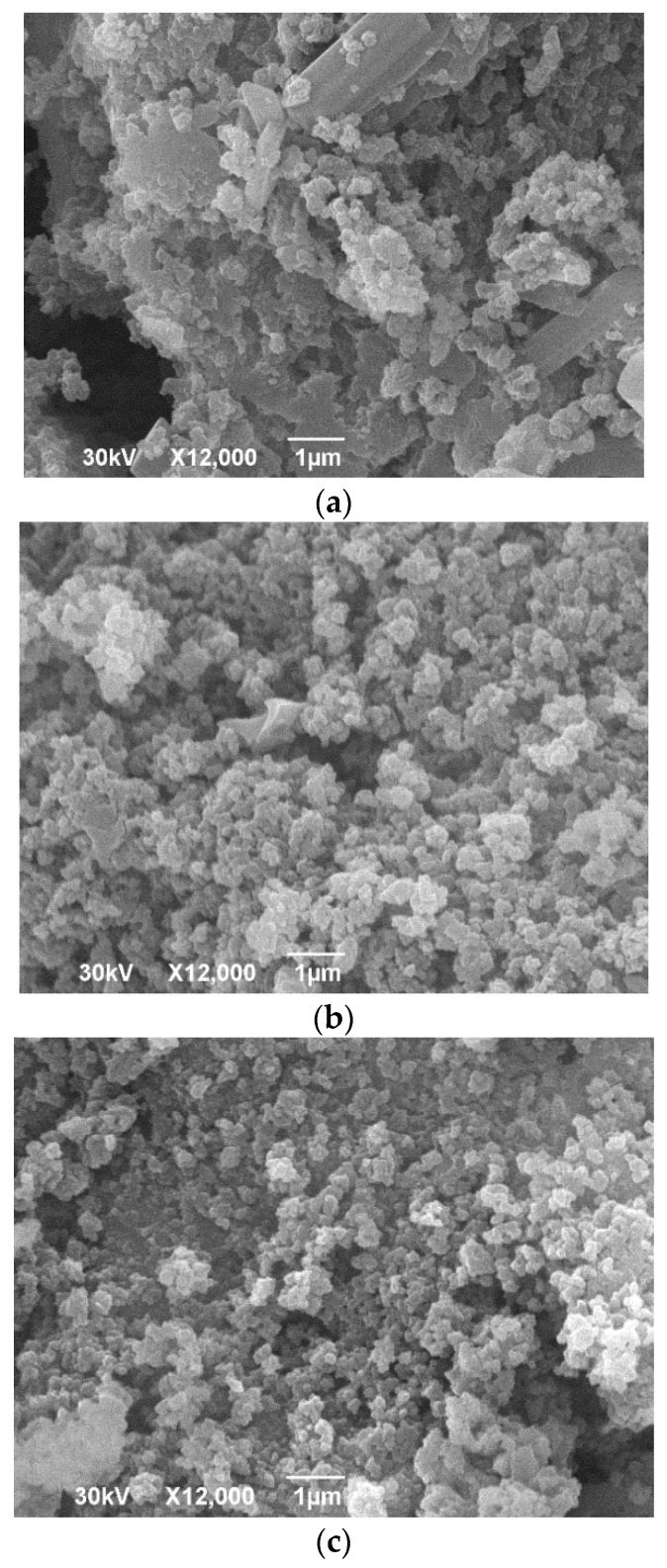
SEM micrographs of GP samples: (**a**) GP, (**b**) GP1, and (**c**) GP3.

SEM results of GP samples are shown in Figure 3. Based on the presented micrograph of the GP sample (Figure 3a), tabular and prismatic particles can be observed, which may present unchanged mineral phases that, due to the geopolymerization process, reveal semi-crystalline morphology. The mineralogical composition can be explained by the process of production of siliceous fly ash, where the glass phase cannot be cooled quickly and uniformly, so there will be united and elongated crystals of minerals in the vitreous phase [54,55]. Among crystal grains of residual minerals from fly ash it is evident, according to Figure 3a, that the rich amorphous polymer phase consists of spherical grains representing a gel-like amorphous matrix where the mineral particles are visible, following the results of XRPD analysis. Figure 3b represents the microstructure of the GP1 sample where spherical particles which differ in shapes and sizes are interconnected, forming aggregates between 50 nm and 1 µm in sizes and forming a homogeneous mass. In some parts, partially transformed grains of silicate minerals covered and immersed into an amorphous matrix can be observed. Formation of a rich amorphous matrix with PVA gel and immersed aluminosilicate minerals in GP1 sample indicate that 1 wt% PVA is fully reacted during the geopolymerization process. Figure 3c shows the microstructure of the GP2 sample where an amorphous geopolymer matrix with irregularly shaped particles with a slightly lower interconnection between them than in GP1 is evident. The addition of 2 wt% PVA in the geopolymer matrix during synthesis leads to the amorphous phase formation. Moreover, a higher percentage of PVA leads to a decrease in the interconnection between the particles, which affects the inhomogeneity of the geopolymer binder matrix. The increase of PVA volume in reaction has an adverse effect on the compactness of the GP2 sample [1].

The EDS semi-quantitative analysis results of the investigated samples are shown in Table 1. The results represent weight percentages of common elements in fly ash and geopolymers. From EDS, the vitreous components of fly ash were dissolved by activator solution to form polymeric gels, except the GP sample, which contained higher percentages of Fe and Ca, and also Al, Si, Na elements which represent plate-like grains with different morphology which stay unchanged during alkali activation process [56]. Due to alkali activation process reaction dissolving vitreous part of fly ash and in case of GP2 sample with 2 wt% of added polyvinyl alcohol there is a higher amount of C and Na. At the same time, contents of Al, K, Ca, and Fe are pretty reduced in correlation to GP and GP1 samples, indicating the creation of polyvinyl aluminosilicate gel. Moreover, lowering the Al concentration results in granular morphology [56,57]. The ratio of Al/Si for GP2 sample results is higher than in GP and GP1 samples, which made it more assertive in terms of its microstructure [58,59].

### 2.4. Mechanical Properties

**Figure 4:**
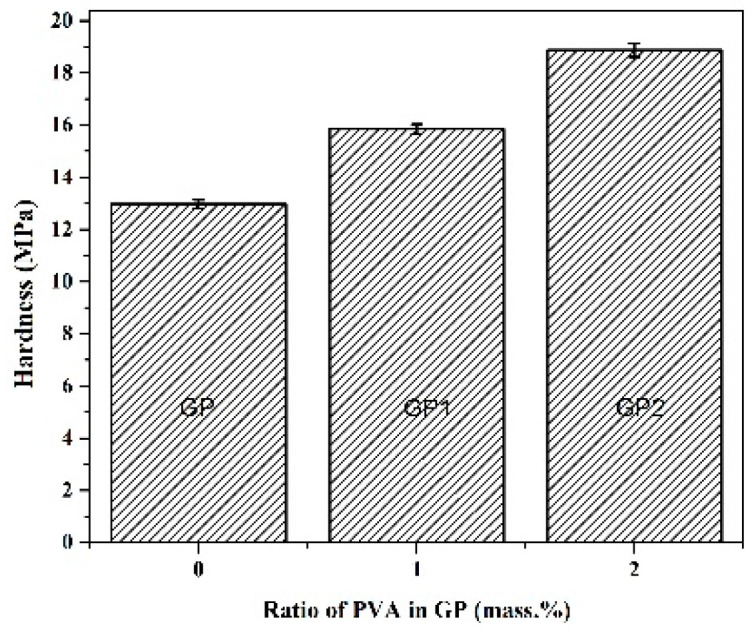
Vickers microhardness of GP, GP1, and GP2 samples.

**Figure 5:**
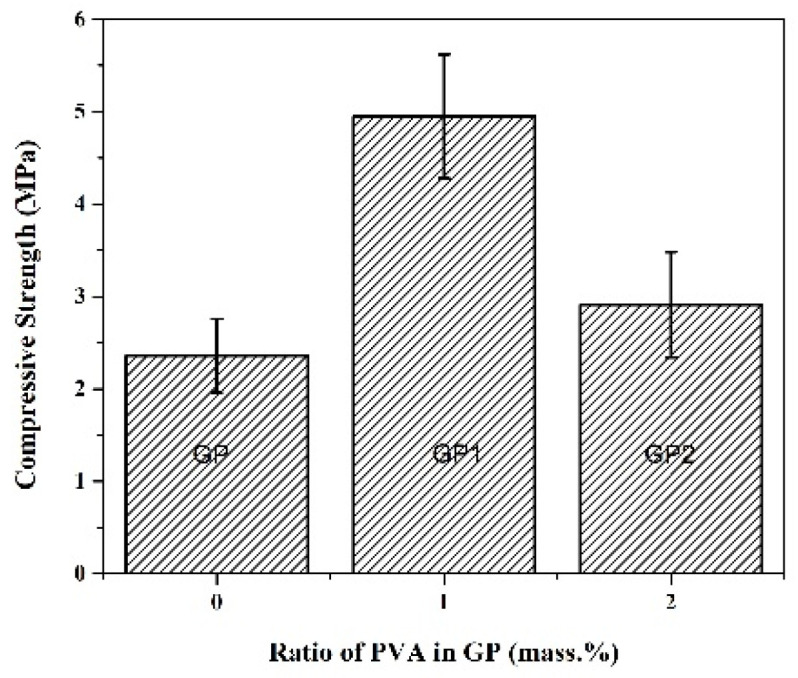
Compressive strength of GP, GP1, and GP2 samples.

The influence of the added amount of PVA on GP mechanical properties is presented in Figure 4. Figure 4 shows the results of the Vickers hardness test of investigated samples. Obtained results show that GP2 with the highest amount of PVA possesses the highest hardness values. Under the compressive load, samples fractured after deformation of 1.78%, 2.16%, 3.18% for PVA ratios 0, 1, and 2, respectively. The data exhibited in Figure 5 shows that addition of PVA in 1% increases the hardness and strength of the matrix; while increasing the amount of PVA causes a decrease in the strength of the matrix. Similar to the literature [60], the compressive strengths of GP increased after loading 1% PVA when cured in dry conditions. This improvement was mainly attributed to the formation of PVA film within the geopolymer matrix. Kim et al. [61] found that the improvement in bond strength after loading PVA seems to arise from suppressing the porous interfacial transition zone and inhibiting calcium hydroxide nucleation on the surface. As the geopolymerization reaction progressed, the PVA precipitated in the geopolymer to form a PVA film. The films can combine with the fly ash alkali-activated product and enhance the connection between the products of this reaction, thereby improving the mechanical properties of the geopolymers. Besides that, the film can become interspersed with the geopolymer, fill the pores and form bridges in the geopolymer gel. Under a load, it can absorb the energy generated by the external force so that the mechanical properties of the geopolymer matrix are improved. The excessive incorporation of PVA led to the formation of heave polymer sheets within the geopolymer matrix. These heavy PVA films might coat FA particles and prevent them from contacting alkali activators. This coated effect of PVA might delay the geopolymerization rate. The lower compressive strength of GP2 over GP1 might be due to its lower geopolymerization rate caused by the coated effect of PVA [61]. The PVA addition in an amount larger than 1% increases the porosity of the sample and weakens the bridges among the matrix, which may cause a decrease in strength (see Figure 3c SEM).

### 2.5. Radiological Properties

Due to the possible use of this type of material in the construction sectors, it is essential to assess the radiological hazard associated with exposure to radiation from ^226^Ra, ^232^Th, and ^40^K [29]. Hazard indexes are used to evaluate radiological threats of the investigated GP, GP1, and GP2 materials. For safety determination of construction materials, hazard indexes should be used only as a screening tool for identifying materials that might be of concern. A typical way of using a certain type of material from the aspect of radiological safety is related to dose assessment [62].

Radiological results of FA01 and its geopolymer products are shown in Table 2. Results represented in Table 2 show that the higher values of specific activities and calculated parameters were obtained for FA01 raw material presented in our previous study [32]. In GP, GP1, and GP2, the most common index (I_γ_) is reduced, and the H_ex_ and H_in_. Since radon and its short-lived products are hazardous to the respiratory organs, the internal exposure to radon and its daughter products is quantified by H_in_ [63]. Considering that H_in_ has the strictest criterion for ^226^Ra activity (185 Bq∙kg^−1^),hence the recommended maximum concentration of ^226^Ra is 200 Bq∙kg^−1^, which shows that *I*_α_ is decreased in all geopolymer samples in comparison to starting fly ash material. Although uranium activity is not of great importance for external exposure, its radiological and toxicological significance is not negligible when it comes to internal exposure by inhalation of fly ash dust. The H_ex_ index is one of the radiation hazard indices that evaluates the radiation dose rate due to the external exposure to gamma radiation from the natural radionuclides in building materials where the value should be less than 1 [64]. In comparison with these propositions, it is evident that the results of our research indicate that the values presented in Table 2 are significantly reduced, especially for GP1 and GP2 samples.

It should be considered that the results of radiological assessments of GP, GP1, and GP2 samples show a decrease of hazard indexes and also activity of radionuclides. Presented detection of lower values of uranium activity is one more positive result of converting fly ash by polymerization process to geopolymer binder. Moreover, the significant decrease of activity concentration of radionuclides in GP samples, especially in GP1 and GP2 samples, is about 50% lower than in starting FA01 material. The results lead to the assumption that the formation of aluminosilicate gel with the addition of Na, Al, Si from activator solution and PVA, on the other hand, leads to reorganization and formation of polymerized aluminosilicate bridges and incorporation and partially stabilization of radionuclides in amorphous matrix leading to reduction of activity concentrations of the main investigated radionuclides. It is evident that the decrease in radionuclide concentration is related to compressive strength, and we assume that this can probably be due to lower porosity, especially in the GP2 sample. These phenomena regarding geopolymer materials radioactivity obtained from FA01 raw material could be recommended as promising binder material as radiological safety material.

## 3. Conclusions

Fly ash from Nikola Tesla power plant FA01 was successfully used as a raw material for geopolymer binder production. The addition of 1 wt% of PVA in synthesized GP1 sample, in geopolymeric gel mixture, and throw alkali activation-synthesis process showed the best phase and microstructural properties and successful incorporation of polyvinyl alcohol in the geopolymer binder material. The amorphous phase is evident on diffractogram as broad background from about 20° 2*θ* to 40° 2*θ* respectively, indicating the formation of geopolymer matrix. This is verified by identifying a weak peak at 1142 cm^−1^ that overlaps with a broad characteristic band (attributed to the Si-O stretching; Si–O–Si symmetric or Al–O–Si asymmetric stretching mode) centered at 1045 cm^−1^. These structures are the building blocks of geopolymers. The results show that the GP1 sample shows the best compressive strength results and satisfactory microhardness values. The excessive incorporation of PVA led to the formation of heave polymer sheets within the geopolymer matrix. This coated effect of PVA might delay the geopolymerization rate and reduce the compressive strength of GP2 relative to GP1. Presented radioactivity results confirm previous research [65,66,67] that after geopolymerization reaction of the raw materials and clays, lower values of gamma emitter activity are detected in the final products of fly ashes. The importance of *I_γ_, H_ex_, H_in_* indexes is about 50% lower in geopolymer samples, especially in GP2 than in FA01. From the point of view of applying this type of material in the construction industry, this conclusion is of immeasurable importance. The obtained results are preliminary and will serve as a reasonable basis for further and more complex research in the field of hybrid organic/inorganic geopolymer materials.

## 4. Materials and Methods

The geopolymer materials are synthesized using FA01 fly ash, which was previously sieved to a grain size of 0.2 mm and then for the synthesis of each geopolymer sample, 25 g fly ash was weighed. Chemical composition of FA01 (19.76% Al_2_O_3_, 61.77% SiO_2_, 6.58% Fe_2_O_3_, 5.32% CaO, 2.06% MgO, 0.51% SO_3_, 0.32% Na_2_O, 1.27% K_2_O) is determined in our previous research [32] according to standard EN 450–1, while LOI-Loss of ignition at 950 °C is 2.06% determined in accordance to standard EN 196–2 [32]. In the obtained results the calculated ratio of SiO_2_/Al_2_O_3_ is 3.13. The referent sample marked as GP is obtained by alkali activation of fly ash. The samples GP1 and GP2 are synthesized by the addition of polyvinyl alcohol-PVA (Merck, p.a. 98%); sample GP1 is made by the addition of 1 wt% PVA, while the GP2 sample is obtained by the addition of 2 wt% of PVA in 25 g of fly ash. In every sample, the ratio of solid to liquid phase was about 1.

The alkali activator was previously prepared from an 8 M solution of NaOH (99%, Analar Normapur) and sodium silicate solution 1.5 S.G. (Fisher Chemical) in a ratio of 1.6 and mixed on a magnetic stirrer for 1 h. The chemical composition of Na_2_SiO_3_ comprised of Na_2_O = 14.7%, SiO_2_ = 29.4%, and water 55.9%, mass ratio.

After the geopolymerization reaction of powder material with activator solution, the samples were poured into cylindrical molds (9 cm × 3 cm) and left to age for 28 days at room temperature.

The X-ray powder diffraction technique was used for the mineralogical analysis of GP, GP1, and GP2 samples. The samples are powdered in porcelain mortar before analysis and put on to Si-monocrystalline sample carrier. The Ultima IV diffractometer was used, equipped with CuKα_1,2_ radiations, using a generator voltage (40 kV) and a generator current (40 mA) with a range from 5° to 80° 2*θ*, the step size was 0.02 and scan rate of 5°/min, using D/TeX Ultra high-speed detector (Rigaku, Tokyo, Japan). The phase analysis was done using PDXL2 (Ver. 2.8.4.0) crystallographic software [68] with the ICDD database [69]. The selected PDF cards numbers are used for phase identification: 00-015-0776-mullite; 00-046-1045-quartz; 01-089-6425-plagioclase (albite); 00-033-0664-hematite.

FTIR analysis was done using the DRIFT technique using Perkin-Elmer FTIR spectrometer Spectrum Two (Waltham, MA, USA). Approximately 5% of powdered samples were dispersed in dried spectroscopic grade KBr (Sigma Aldrich, p.a., St. Louis, MO, USA) with a reflective index of 1.559 and particle size of 5–20 μm. The spectra were scanned in the mid-infra-red region from 4000 to 400 cm^−1^.

SEM-EDS analysis was performed using JSM-6390 (LV JEOL, Tokyo, Japan). The samples were Au coated before the examination. EDS analysis was done on the whole scanning surface selected for study (area 15 × 15 μm).

For compressive and hardness tests, samples were mechanically shaped into cylindrical specimens (24 mm in diameter and 38 mm in height), ground and polished. Three pieces of each GP, GP1, and GP2, were used to calculate the average values. The compressive test was performed with a 2 mm/min loading rate, using Instron 1185 universal testing machine (Norwood, MA, USA). Vickers macro hardness tests were performed using Buehler Hardness Tester (Leinfelden-Echterdingen, Germany) under the load of 9.8 N, with dwell 5 s, and hardness values are obtained from an average of 5 indents.

Radiological assessments and the radionuclide activity were determined by following the emission of gamma-ray on HPGe detector (Canberra, Meriden, CT, USA) (relative efficiency 50%, resolution 1.89 keV at 1332 keV), and the fly ash sample was investigated that was left for at least 28 days in PVC cylindrical containers (250 mL) sealed with beeswax, due to the establishment of radioactive equilibrium between radon and its progenies. The radioactive balance is achieved in the prepared samples of geopolymer GP, GP1, and GP2, but in the vial geometry. An efficiency calibration was performed using the secondary reference materials in the 250 mL PVC cylindrical containers and the vial geometry produced from the radioactive solution 1035-SE-40844-17 issued by the Czech Metrological Institute, Inspectorate for Ionizing Radiation, which contained ^210^Pb, ^241^Am, ^57^Co, ^60^Co, ^137^Cs, ^139^Ce, ^85^Sr, ^109^Cd, ^88^Y and ^51^Cr, and traceable to the BIPM.

## Figures and Tables

**Table 1:** EDS results of synthesized GP, GP1, and GP2 samples.

Element, (wt%)	GP	GP1	GP2
C	17.41	17.89	24.57
O	43.73	45.65	41.13
Na	7.70	6.98	7.96
Al	4.92	5.33	3.75
Si	19.73	19.37	19.16
K	0.71	0.56	0.49
Ca	2.35	2.19	1.19
Fe	3.43	2.03	1.75

**Table 2:** Radiological results of FA01 and investigated samples.

Activity Concentration [Bq∙kg^−1^]				
Radioisotope	FA01 [32]	GP	GP1	GP2
^226^Ra	101 ± 4	50 ± 4	45 ± 5	37 ± 6
^232^Th	81 ± 6	58 ± 9	45 ± 9	37 ± 8
^40^K	387 ± 26	182 ± 31	169 ± 30	160 ± 30
^137^Cs	<0.1	<2	<2	<2
^238^U	140 ± 20	<50	<50	<55
^235^U	7.8 ± 0.6	4.4 ± 0.9	<3	<3
*I_γ_*	0.87	0.51	0.43	0.36
*H_ex_*	0.67	0.39	0.33	0.28
*H_in_*	0.94	0.52	0.42	0.38

## Data Availability

No new data were created or analyzed in this study. Data sharing is not applicable to this article.
